# pHEMA: An Overview for Biomedical Applications

**DOI:** 10.3390/ijms22126376

**Published:** 2021-06-15

**Authors:** Mina Zare, Ashkan Bigham, Mohamad Zare, Hongrong Luo, Erfan Rezvani Ghomi, Seeram Ramakrishna

**Affiliations:** 1 Center for Nanotechnology and Sustainability, Department of Mechanical Engineering, National University of Singapore, Singapore 117581, Singapore; erfanrezvani@u.nus.edu; 2 Institute of Polymers, Composites and Biomaterials—National Research Council (IPCB-CNR), Viale J.F. Kennedy 54—Mostra d’Oltremare pad. 20, 80125 Naples, Italy; ashkan.bigham@ipcb.cnr.it; 3 Health Science Center, Xi’an Jiaotong University, Xi’an 710061, China; mikayilzare1997@gmail.com; 4 National Engineering Research Center for Biomaterials, Sichuan University, Chengdu 610064, China; hluo@scu.edu.cn

**Keywords:** biomedical application, antimicrobial strategies, cancer therapy, contact lens, ocular drug delivery, pHEMA, tissue engineering and regenerative medicine

## Abstract

Poly(2-hydroxyethyl methacrylate) (pHEMA) as a biomaterial with excellent biocompatibility and cytocompatibility elicits a minimal immunological response from host tissue making it desirable for different biomedical applications. This article seeks to provide an in-depth overview of the properties and biomedical applications of pHEMA for bone tissue regeneration, wound healing, cancer therapy (stimuli and non-stimuli responsive systems), and ophthalmic applications (contact lenses and ocular drug delivery). As this polymer has been widely applied in ophthalmic applications, a specific consideration has been devoted to this field. Pure pHEMA does not possess antimicrobial properties and the site where the biomedical device is employed may be susceptible to microbial infections. Therefore, antimicrobial strategies such as the use of silver nanoparticles, antibiotics, and antimicrobial agents can be utilized to protect against infections. Therefore, the antimicrobial strategies besides the drug delivery applications of pHEMA were covered. With continuous research and advancement in science and technology, the outlook of pHEMA is promising as it will most certainly be utilized in more biomedical applications in the near future. The aim of this review was to bring together state-of-the-art research on pHEMA and their applications.

## 1. Introduction

Poly(2-hydroxyethyl methacrylate) or pHEMA is a biocompatible, optically transparent, hydrophilic, and non-degradable polymer. Moreover, it resists to crack propagation leading to a high load bearing ability [1,2,3,4]. pHEMA is known to have a glass transmission (t_g_) temperature and density in the range of 358–393 K, and 1.15–1.34 g mL^−1^ respectively [5]. pHEMA is a biomaterial and a non-toxic polymer of the toxic monomer HEMA [6]. pHEMA was initially studied and invented by Otto Wichterle and Drahoslav Lim in the development of modern soft hydrogel contact lenses in 1960 [2]. pHEMA was first used as an orbital implant, after which Wichterle and Lim thought the biological and mechanical properties of pHEMA made this biomaterial suitable for developing contact lenses [7]. Throughout the years, advancement in research and development has allowed pHEMA to be utilized in a broad range of biomedical applications. These applications include soft contact lenses, bone tissue regeneration, ocular drug delivery and controlled drug delivery systems, artificial corneas, artificial skin, wound dressings, breast augmentation, catheters, intrauterine inserts, and prosthesis [8,9,10,11]. Based on the materials, the contact lenses are classified into soft and hard. The soft contact lenses made of pHEMA are flexible and oxygen permeable that allows oxygen to pass through the cornea whereas the hard contact lenses (PMMA) are made of rigid gas permeable materials. FDA categorized the soft material contact lenses into four groups including nonionic (low water content, <50%), nonionic (high water content, >50%), ionic (low water content, <50%), and ionic (high water content, >50%) [12]. Table 1 represents the advantages and disadvantages of pHEMA applications.

**Figure 1:**
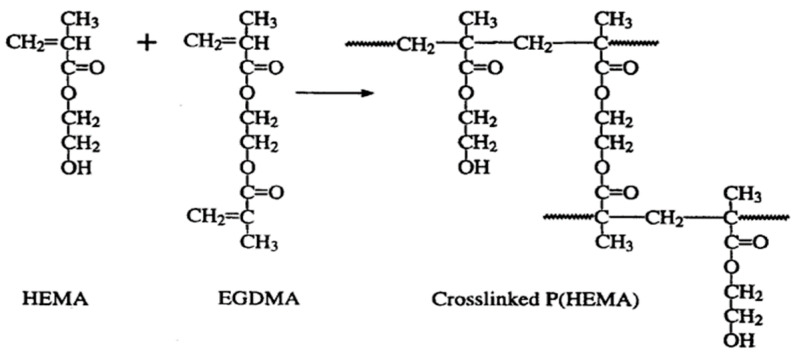
A schematic representing the synthesis of pHEMA hydrogel from copolymerization of HEMA with other co-monomers, (EGDMA, ethylene glycol dimethacrylate). Reprinted from [27] with permission from Taylor & Francis.

Hydrogels are highly hydrophilic polymeric networks that have gained rapid popularity because of their superior biocompatibility, processability, and their similarity in characteristics to a cell’s native extracellular matrix [17,18,19]. pHEMA is a biocompatible, non-biodegradable and optically transparent hydrophilic polymer that becomes swollen and forms a hydrogel with the absorption of water or biological fluids due to its hydrophilic pendant group. In its dry state, pHEMA exists as a hard and brittle material. Upon swelling, it becomes flexible and soft and can be cut easily. Transparent pHEMA-based hydrogels allow liquid and oxygen diffusion and are highly permeable to small molecules [20,21]. pHEMA-based hydrogels also exhibit superior biocompatibility, cytocompatibility, hydrophilicity, and low friction coefficient. The mechanical (tensile strength and Young’s modulus) and optical (light transmission) properties, oxygen permeability, and water absorption of pHEMA-based hydrogels can be altered by different techniques of polymerization and copolymerization, different cross-linking rate etc. [8,21]. Thermal and photochemical polymerization are the techniques by which HEMA can be either polymerized or copolymerized. Homogenous pHEMA hydrogels are obtained when HEMA is polymerized in the exposure of small amounts of water. If the water content goes beyond 60% by volume, the phase separation takes place because the chains with more than 20 repeat units in length form. Growing in the chains length results in more phase separation followed by forming the polymer droplets. Next, the aggregation of droplets leads to the formation of porous hydrogel [22,23]. Based on the polymerization technique, the porous pHEMA can be applied in different applications. Regarding to the great physicochemical and biological properties of pHEMA, it has been applied as an implant rooting in its inertness and non-degradability. Moreover, HEMA contains some impurities related to cross-linkers, such as ethylene dimethacrylate making the polymer insoluble in water. Therefore, a great effort has been made to yield degradable pHEMA hydrogels through copolymerization of HEMA with different hydrophilic methacrylates. Another way to improve the polymer’s degradability is to lower the molecular weight below 2500 Da [24,25]. The studies revealed controlled synthesis approaches for pHEMA, which allows for the synthesis of block copolymers with highly controllable material properties. This is highly relevant for more advanced pHEMA applications [26]. Figure 1 represents the schematic of the free radical crosslinking polymerization of pHEMA.

As pHEMA is expansively applied in ocular-related applications, contamination of contact lenses, lens cases, and lens care solutions are risk factors for developing ocular infections, including microbial keratitis (MK). The severe visual consequence of MK is corneal perforation. The resistant biofilm formation on the surface of contact lenses is due to adherence and colonization of bacteria, such as *Staphylococcus aureus*, *Staphylococcus epidermidis*, and *Pseudomonas aeruginosa* [28,29,30]. Hence, antimicrobial strategies to develop permanent or long-term contact lenses are of great importance.

In this review, we covered potential applications of pHEMA in biomedical engineering. In this regard, the potential of pHEMA in bone tissue regeneration, wound healing, and cancer therapy is discussed. It is noteworthy that, as pHEMA is expansively assessed for ocular-related applications, considerable attention is devoted to pHEMA in this field. Moreover, the antimicrobial strategies for pHEMA hydrogel contact lenses and ocular drug delivery are presented. In the end, the future outlook of pHEMA in the field will be introduced.

## 2. Bone Tissue Regeneration

As bone is naturally composed of both organic and inorganic compounds, a combination of polymers and ceramics to mimic the bone’s structure has always been a target. The logic behind this combination is to bring the flexibility and rigidity of polymers and ceramics into a package, respectively [31]. However, it is not an easy task, as essential requirements for a bone graft must be met, including biocompatibility, biodegradability, desirable mechanical properties based on the host tissue, osteoconductivity, osteoinductivity, and porosity [32]. Although pHEMA is endowed with great potential in biomedical engineering, it is a non-degradable polymer and, thus, many attempts have been made to address this issue. In an attempt to take the advantages of pHEMA in bone regeneration, it was used in combination with hydroxyapatite as a hybrid biocomposite hydrogel. Hydroxyapatite is regarded as the inorganic phase of bone and a gold standard in bone tissue engineering. Therefore, it is not surprising to see its widespread use in combination with different materials [33,34,35]. The biocomposite hydrogel exhibited elastomeric properties and, thus, the composite could be bent, cut, and machined into a desirable shape. However, this composite was non-degradable, restricting its application in bone tissue regeneration [36]. A probable solution to that problem was introduced through modifying the pHEMA/hydroxyapatite biocomposite with a degradable cross-linker. The addition of cross-linker (dimethacrylated poly(lactide-b-ethylene glycol-b-lactide) was synchronized with the hydrolytic degradation of composite and based on the cross-linker’s chain length, molecular weight, and concentration, the degradation rate can be altered [37]. In another study accomplished by the same group, pHEMA was combined with the mixture of hydroxyapatite and beta tricalcium phosphate, but a different cross-linker (N,O-dimethacryloyl hydroxylamine) had been used. Moreover, a co-monomer (acrylic acid) was introduced into the composite. The type and concentration of co-monomer and cross-linker were evident to increase the degradation, while increasing in the bioceramic content decreased the weight loss; this phenomenon was attributed to the strong interaction between the ceramic particles and the anionic acrylic acid [38]. A ternary bone scaffold for bone regeneration was reported elsewhere, composed of polyurethane/pHEMA/cellulose nanowhiskers. Notably, pHEMA was cross-linked with acrylic-urethane in situ in the presence of other phases. The increasing in the nanowhiskers was accompanied with a significant improve in the physicochemical and biological properties of scaffolds [13]. Besides using structural modifiers to make pHEMA degradable, blending this polymer with a degradable one would be another solution to its non-degradability. Polycaprolactone (PCL) is known as an FDA approved biocompatible and degradable polymer with different applications in biomedical engineering. The main drawbacks of PCL, such as poor bioactivity and cell attachment, attributed to its hydrophobic nature [39]. Through a recent study, pHEMA was blended with PCL to obtain a degradable and hydrophilic hybrid. Moreover, to reinforce the mechanical properties of hybrid, a ceramic composed of hydroxyapatite and calcite was incorporated. Although no comparison was made between pure PCL and PCL/pHEMA, it is observable that the hybrid without the ceramic phase had about 20% water uptake and desirable cell attachment; moreover, the water contact angle was 89°. The obtained results implied that blending of both polymers culminated in the compensation of their restrictions—poor degradability and wettability [40]. As showcased, when it comes to designing a bone graft or scaffold, many requirements must be met. Tailorable physical and chemical properties of pHEMA would provide an opportunity to alter the final properties of bone graft based on the need and intended application.

## 3. Wound Healing

The skin is formed mainly by three distinct layers, each of which has its own structure and function. The outer layer of skin is called the epidermis; the main duty of this layer is to protect the internal parts of the body from any potential danger from the outside, such as bacteria, contamination, etc. The protective role of the epidermis is accomplished through a ‘’brick and mortar’’-like structure, in which corneocytes are bricks surrounded in non-polar lipids. The next layer is the dermis, mainly responsible for the mechanical support of skin tissue through the existence of extracellular matrix components. Moreover, different types of cells and compartments are located here, among which, fibroblasts outnumber the others as they can play a key role in the wound healing process and remodeling. The third layer is the hypodermis, which has important functions in the skin as the heat insulator and shock absorber [41].

Based on the depth, layer, and repair rate, wounds can be different from one another. A full-thickness refers to a wound when all three layers are involved, and even deeper tissues, such as muscles and bone. When it comes to healing rate, the wounds are known as acute and chronic [42]. The former has a predictable time to heal completely and the time differs based on the wound severity, depth, etc. It complicates matters when the wound is diagnosed as chronic, because, in this case, the healing process gets stuck in one of the healing stages, hampering the process from completion. There are many reasons causing this process to prolong—immune-related diseases, diabetes, infection, age, etc. These cases require more hospitalization and effective therapies [43].

**Figure 2:**
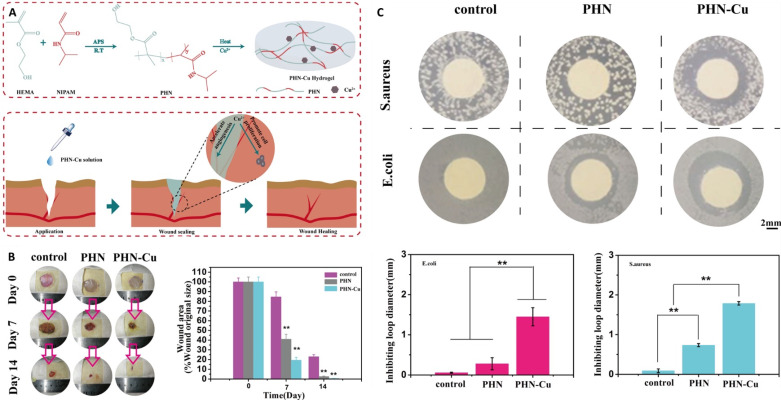
A schematic showing the preparation of thermosensitive polymer followed by wound healing (**A**); the in vivo results of different samples when they were applied on the wounds for 14 days (**B**); the antibacterial activity of samples against both *Staphylococcus aureus* and *Escherichia coli* bacteria (**C**); ammonium persulfate (APS), radical transformation (RT), poly-(HEMA-co-NIPAM) (PHN), reprinted from [11] with permission from Wiley. ** denotes *p* < 0.01.

Pivotal properties for ideal wound dressing include absorption of detrimental exudates, keeping the wound’s moisture at an appropriate level, non-toxic nature, highly permeable to oxygen, anti-infection, and transparency. Because of high biocompatibility and low thrombogenicity, pHEMA has been widely used in ocular-related applications. pHEMA hydrogels with a water content in the range of 32–43% are receiving considerable attention in wound healing applications. One recent study combined the great potential of pHEMA with bacterial cellulose whiskers, and silver, to design a multifunctional wound dressing. The cross-linking polymerization of HEMA was accomplished when the whiskers were present. The whiskers significantly improved both water uptake and transparency simultaneously. The silver ions through the hydrogel showed acceptable antibacterial activity against both Gram-positive and Gram-negative bacteria. Besides the positive properties of prepared hydrogel, it was transparent, allowing to see the wound beneath the dressing and tracking healing process. Based on pHEMA, a thermosensitive polymer composed of pHEMA/polyNIPAM/Cu was developed through free-radical polymerization for wound healing applications. The sol was “turned out” to undergo gelation when the temperature rose to 29 °C. Designing such thermosensitive material is of particular interest because it can be injected through complex shape wounds, followed by being gelled. Therefore, shape processing and fabrication can be removed, making the prepared wound dressing both cost-effective and efficient. The addition of Cu to the polymer matrix culminated in both antibacterial activity and angiogenesis. The in vivo results implied that the ternary polymer decreased the healing time significantly [11]. Figure 2 shows how the thermosensitive polymer was prepared and used as a wound dressing. Based on the water uptake of pHEMA, the wound dressing composed of this polymer benefited from absorbing the exudates, besides its great physicochemical and biological properties. Moreover, the transparency of pHEMA allows medical practitioners to easily track the wound healing process.

## 4. Cancer Therapy

**Figure 3:**
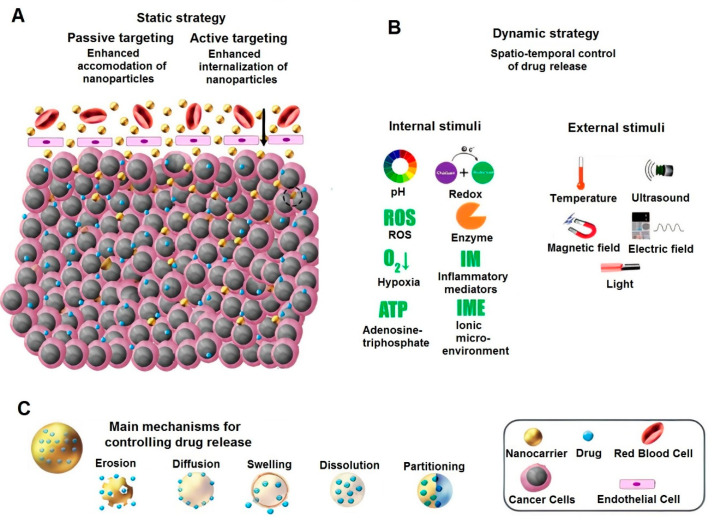
A schematic showing different approaches toward drug delivery for cancer therapy—static (**A**), dynamic (**B**), and main mechanism for controlling drug release (**C**). Reprinted from [49] with permission from Elsevier.

When referring to diseases with the highest causes of mortality—cancer is second, right after cardiovascular diseases [44]. The most common approaches to treat cancer are chemotherapy and radiation, which are capable of killing cancerous cells, but with catastrophic side effects for healthy cells. Newer methods mainly focus on two points: one involves decreasing the detrimental effects of chemotherapeutic drugs on healthy cells and the other involves delivering the “cargo” to the targeted site. To achieve these aims, researchers have published a number of studies on these approaches in recent years. Different drug delivery systems based on polymers, metals, ceramics, and composites were reported to not only present a controlled release property over an intended time period, but also be smart and responsive to disparate stimulants [45,46,47]. An injection of drug molecules, with/without a carrier directly through the blood stream, is a common method in cancer therapy. When the drug molecules or drug-loaded nanoparticles enter the body, they interact with endothelium in blood vessels to reach to the tumor site. If the nanocarrier is not responsive to any stimulant, its anticancer activity would fall into a passive targeting approach, which is mainly based on enhanced permeation and retention. Moreover, the release rate is based on the structure and design of the nanocarrier. Regarding the type of material, structure, and physical and chemical properties, they can release the drug through different mechanisms. On the other hand, active targeting uses some modifiers on the nanoparticle surface to “exactly” attach, this is followed by internalizing into the cancerous cell [48]. Moreover, there are other active targeting approaches based on internal and external stimulants. The internal-based stimuli responsive carriers are activated when they are exposed to the specific stimuli, such as pH, redox, ROS, etc. [49,50]. The external responsive carriers can be controlled or activated through different techniques. For instance, magnetic nanoparticles respond to an external magnetic field to either reach an intended site and/or generate heat to eradicate the cancerous cells through hyperthermia [51]. Figure 3 exhibits the different drug delivery mechanisms towards cancer therapy.

**Figure 4:**
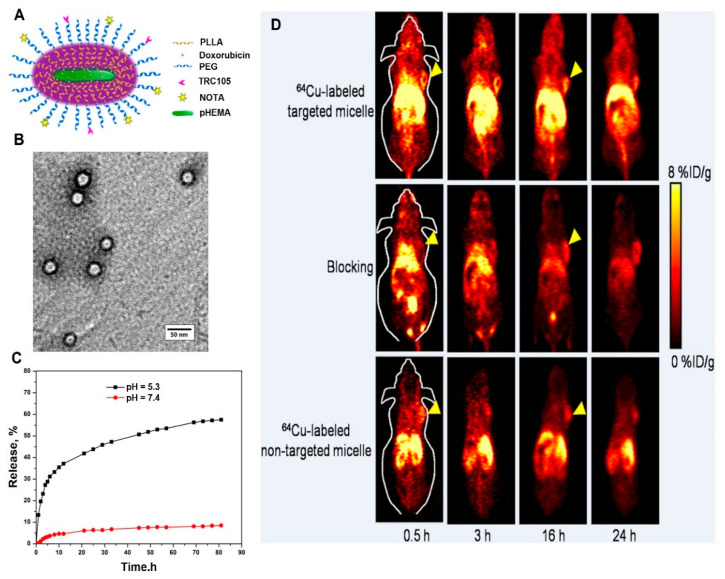
A schematic of the micelle (**A**); TEM micrograph of the micelle (**B**); the release rate of doxorubicin-loaded micelle at physiological and acidic mediums (**C**); the positron emission tomography images of 4T1 tumor-bearing mice treated with ^64^Cu-labeled targeted, non-targeted micelles, and targeted micelles with a blocking dose of TRC105 at different time intervals (**D**). Reprinted from [55] with permission from American Chemical Society.

Regarding the tailorable physical properties of pHEMA through formulation chemistry, it is applied as both a passive and targeted drug delivery system, either solely or in combination with other compounds. A passive delivery system, based on pHEMA nanoparticles, was developed, and 5-Fluorouracil was used as an antitumor drug. The main aim was to design a swelling controlled drug delivery system. The pHEMA nanoparticles in the range of 100–300 nm with 6–23% drug loading efficiency were successfully obtained. Ethylene glycol dimethacrylate was used as the cross-linker in this study; as it turned out, the increase of the HEMA monomer caused a decrease in the drug release rate, while an increase in the cross-linker resulted in vice versa [52]. The same group again synthesized the pHEMA nanoparticles through the same method, but used doxorubicin as a well-known anticancer drug. Moreover, 28% of the drug entrapment was obtained; the release rate could be altered by changing in the experimental parameters. Different from their previous study—focusing on 5-Fluorouracil—the doxorubicin-loaded nanoparticles exhibited a fast release at acidic medium, while the drug carrier was not supposed to be pH-responsive. The reason can be related to the cationic charge of doxorubicin as it altered the surface charge of nanoparticles resulting in an increase in the swelling properties at a lower pH [53]. A 5-Fluorouracil–loaded carrier composed of natural deep eutectic solvents and pHEMA was synthesized for cancer therapy. The physical structure was found porous with great potential for drug loading and sustained release. As expected, the encapsulation efficiency was approximately 77%, with a controlled release rate up to 130 h. The unloaded carrier showed no toxicity against HeLa cancer cells, whereas the anticancer-loaded sample effectively suppressed their proliferation [22]. The mentioned studies conducted research on hydrophilic drugs mostly, but via a report, curcumin as a hydrophobic drug was encapsulated through pHEMA. The drug loading efficiency improved through gelled ionic liquid, obtaining a loading of 26.4%. The in vitro cell toxicity was assessed through ovarian cancer cells. The results implied that the capability of curcumin-loaded nanoparticles against the cancer cells was higher than pure curcumin [54]. To improve pHEMA functionality, it was combined with other polymers to yield a multifunctional drug delivery system for simultaneous diagnosis and therapy. A unimolecular brush-like micelle composed of pHEMA-poly(L-lactide)-poly(ethylene glycol) was designed for cancer therapy. The poly(ethylene glycol) was conjugated with TRC105 and 1, 4, 7-triazacyclononane-N, N′,N-triacetic acid for positron emission tomography imaging. Doxorubicin was chosen as the anticancer drug and the release studies revealed that the drug liberation was pH-dependent. In vivo studies showed a higher accumulation of nanoparticles in tumors treated with ^64^Cu-labeled targeted micelles. Based on the results, the nanocarrier has a promising potential in theranostic-based cancer therapy [55]. Figure 4 indicates the structural, drug delivery, and imaging potential of the multifunctional micelles.

**Figure 5:**
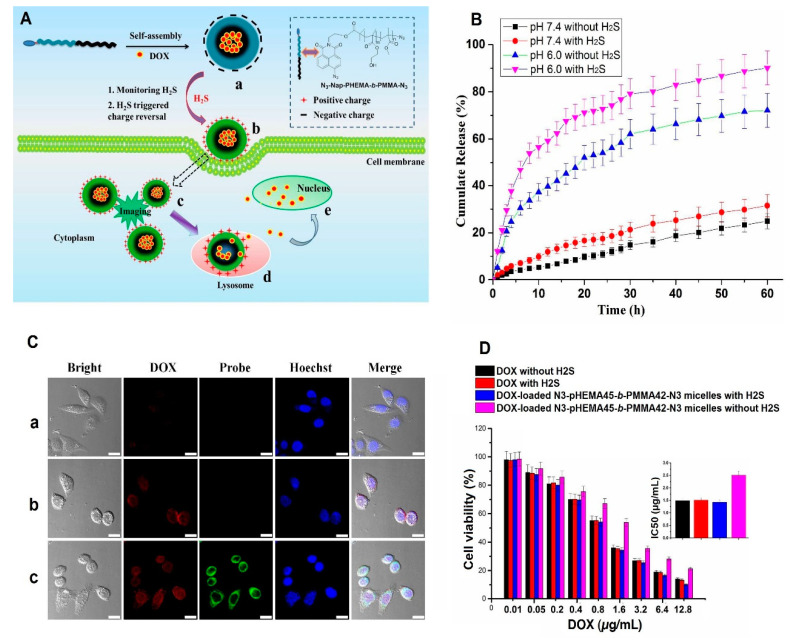
The mechanism of the drug-loaded hydrogen sulfide-responsive micelles when exposed to the stimulant is depicted schematically (**A**); the drug release rates of the drug-loaded N3-Nap-pHEMA-b-PMMA-N3 sample at acidic and physiological environments, with/without being exposed to hydrogen sulfide (**B**); the images of HeLa cells treated with doxorubicin (**a**), drug-loaded N3-Nap-pHEMA-b-PMMA-N3 without hydrogen sulfide (**b**), and drug-loaded N3-Nap-pHEMA-b-PMMA-N3 with hydrogen sulfide (**c**), taken by confocal microscopy (scale bar = 20 μm) (**C**); the cell viability (%) of drug and non-loaded samples, with/without being exposed to hydrogen sulfide (**D**). Reprinted from [16], with permission from the American Chemical Society.

**Figure 6:**
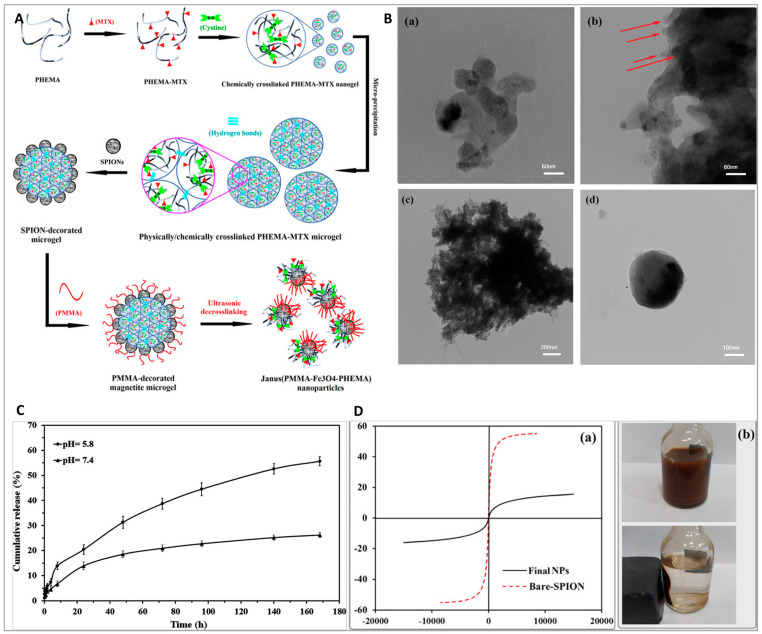
A schematic exhibiting the synthesis procedure of pHEMA-PMMA-magnetite (Fe_3_O_4_) (**A**); the TEM micrographs of the cross-linked pHEMA nanogel (chemically) (**a**), the cross-linked pHEMA microgel (physically and chemically) (**b**), Fe_3_O_4_-coated cross-linked pHEMA-methotrexate (**c**), pHEMA-PMMA-Fe_3_O_4_ (Janus) (**d**) (**B**); the release trends of methotrexate from pHEMA-PMMA-Fe_3_O_4_ nanoparticles (**C**); the magnetic hysteresis loops of bare Fe_3_O_4_ and final pHEMA-PMMA-Fe_3_O_4_ nanoparticles (**a**) plus separation of pHEMA-PMMA-Fe_3_O_4_ nanoparticles in water by a magnet (**b**) (**D**). Reprinted from [15], with permission from the American Chemical Society. (Red arrow: in Figure (**b**) show coagulation of cross-linked pHEMA nanogel).

Nowadays, smart nanomaterials are at the forefront of every field of study as their efficiency and applicability are definitely evident compared to the conventional materials. As briefly discussed in the previous paragraph, smart nanoparticles in cancer therapy can be designed in a way to respond to a specific stimulus, e.g., temperature, pH, enzyme, redox, etc. In the case of cancer therapy, cancerous cells and tumor microenvironments have some specific features by which a smart drug delivery system can be triggered [47]. For instance, cancerous cells generate high amounts of hydrogen sulfide, which can be used against cancerous cells to yield a smart delivery system; this stimulant’s advantage was taken to synthesize a smart drug delivery system for cancer diagnosis and targeted therapy. A series of micelles composed of N-(2-hydroxyethyl)-4-azide-1,8-naphthalimide ended amphiphilic diblock copolymer poly(2-hydroxyethyl methacrylate)-block-poly(methyl methacrylate) (N3-Nap-pHEMA-b-PMMA-N3), loaded with doxorubicin, was designed and prepared. In the presence of hydrogen sulfide, the azido groups on the micelles reduced amid groups, culminating in green fluorescence and charge reversal. Moreover, the drug-loaded sample showed a faster release rate when exposed to hydrogen sulfide. Fluorescence imaging revealed that the drug-loaded micelles accumulated in the tumor’s site selectively [16]. Figure 5 exhibits the applicability potential of hydrogen sulfide-responsive drug-loaded and non-loaded N3-Nap-pHEMA-b-PMMA-N3 micelles in cancer detection and therapy. It is well-known that the tumor microenvironment has an acidic pH, and by having a pH–responsive delivery system that is protonated in an acidic medium, the cargo is liberated when the carrier reaches the tumor’s microenvironment; thus, the hazardous effects of chemotherapeutic drugs on the healthy cells can be hampered [56,57]. Considering the swelling behavior and hydrophilic nature of pHEMA, it was used for improving the swelling behavior of N, N-dimethylaminoethyl methacrylate as a cationic polymer [58]. A pH-sensitive nanohydrogel delivery system was designed based on the combination of those polymers. At the physiological environment (pH = 7.4), no significant drug release was observed, whereas at acidic mediums, the faster release was evident. Therefore, it can alleviate the drawbacks of naked chemotherapeutic drugs through releasing the cargo in the cancerous environment [59]. Nonetheless, pHEMA on its own is considered a stimuli-responsive polymer. pHEMA with the degree of polymerization in the range of 20–35 was shown to have thermo-responsive capability (cloud point changes from 28 °C to 39 °C (pH = 6.5)) [60]. An external-based stimuli-responsive nanocarrier composed of hydrophobic PMMA, hydrophilic pHEMA, and magnetite nanoparticles was designed for cancer therapy. Methotrexate was first grafted on the low molecular pHEMA followed by making a covalent bond between magnetite nanoparticles and the drug-grafted polymer. The main aim was to achieve Janus nanoparticles. At the final step, through the unreacted side of grafted magnetite nanoparticles, the MMA monomers were grafted, and an ultrasound process was then carried out. Figure 6 indicates the synthesis process (A), the TEM micrographs of materials obtained at each step of the synthesis process (B), the release rate (C), and the magnetic property of nanoparticles (D). The TEM micrograph of the Janus nanoparticle (Figure 6B(d)) clearly exhibits that there are two distinct areasvisible proving the successful synthesis of Janus structure. The obtained drug delivery system was turned out as a double-stimuli-responsive carrier—pH and magnetic—with great potential for cancer therapy.

## 5. Soft Contact Lenses

### 5.1. Vision Correction

Contact lenses are ocular prosthetic devices widely used to correct vision. They can also be used for aesthetic or therapeutic purposes. A contact lens is a thin, curved plastic disk designed to be worn directly on the cornea for the correction of vision disorders of the eye. Like eyeglasses, contact lenses correct vision disorders caused by refractive errors whereby the eye is unable to refract light properly onto the retina, resulting in blurred images perceived by the brain [61,62]. Common types of refractive errors include astigmatism, myopia, hyperopia, and presbyopia. Besides blurred vision, refractive errors may also result in squinting, headaches, or eye strain. Contact lenses help to improve vision by correcting the light refraction path into the eye, thereby focusing light better on the retina. Unlike eyeglasses, contact lenses have the advantage of providing maximum peripheral vision. This makes them more convenient and especially useful in sports. They are also useful in correcting vision when there is a huge difference in the power of each eye [63].

pHEMA-based hydrogels are especially suitable in the fabrication of soft contact lenses. Besides being transparent, soft, and flexible, pHEMA-based hydrogels also exhibit superior biocompatibility, high oxygen permeability and sufficient mechanical strength [64]. Being soft and flexible aid in the comfort of wearers of soft contact lenses as opposed to rigid lenses, while the high oxygen permeability helps prevent adverse clinical events associated with corneal hypoxia. The mechanical strength of pHEMA-based hydrogels also ensures that soft contact lenses are reasonably durable [62,65,66,67].

Three-dimensional (3D) printing of contact lenses for vision correction is currently in the research and development phase. In 2016, Johnson & Johnson Vision Care launched a collaboration with HP, Inc. to develop 3D printable contact lenses. Johnson & Johnson researchers are working with HP’s 3D printing business team to utilize smart and 3D printing technology to develop 3D printable contact lenses that can be personalized according to the needs of the patients and can be used to correct vision, reduce glare, eyestrain, and relieve eye allergies [68,69,70]. Therefore, 3D printing of high precision contact lenses for vision correction and ocular drug delivery is potentially feasible in the future.

### 5.2. Ocular Drug Delivery

Delivery of therapeutic drugs to the eyes through utilizing contact lenses fabricated from pHEMA-based hydrogel is a promising method to treat eye infections and disorders. Topical ocular drug administration can be used to treat superficial eye infections, such as conjunctivitis, blepharitis, and keratitis and intraocular disorders through corneal absorption such as uveitis and glaucoma [18,71].

**Figure 7:**
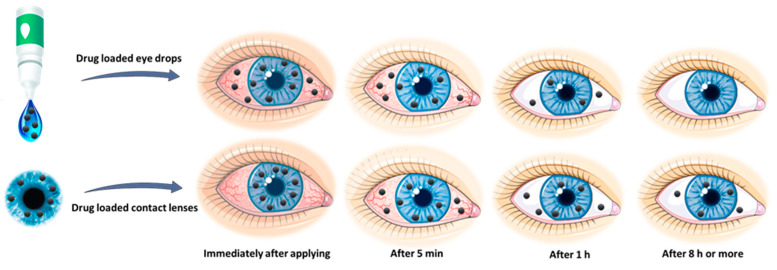
Schematic representation depicting the difference in drug release time using contact lenses as compared to the conventional topical formulation.

Owing to the anatomical and physiological constraints of the eye, a major challenge of administering medication to the eye includes delivering an optimal concentration of the medicine to the active site for a necessary residence time so as to maximize the therapeutic effects. Drug delivery to the eye using drug-loaded hydrogel contact lenses enables a controlled and extended release of an optimal drug concentration by diffusion through the hydrogel system. This may improve ocular treatment efficacy by enabling higher bioavailability, prolonged residence time, lower systemic absorption, and improved patient compliance with dosage regimens [72]. Contact lenses loaded with drugs can provide treatment for ocular diseases and refractive errors at the same time, while patients without refractive error may utilize neutral lenses. Figure 7 displays the difference in drug release time using contact lenses and eye drops.

Soft contact lenses fabricated from pHEMA-based hydrogels are especially suitable for ocular drug delivery. pHEMA-based hydrogels are soft, flexible, and highly oxygen permeable, and provide safety and comfort to patients during ophthalmic treatments. When a drug-loaded pHEMA-based hydrogel lens is applied onto the cornea of the eye, a thin film of tear is created, which gets trapped between the hydrogel lens and the cornea, resulting in the release of drugs through diffusion. The turnover duration of the tear film is longer compared to a normal tear film, resulting in a prolonged precorneal residence time. This results in higher bioavailability and a more accurate dose of the drug being delivered to the active site [61,73]. With the use of pHEMA-based lenses, patient compliance to dosage regimen can be more assured. In addition, the cost of ocular drug delivery by means of contact lenses is lower than repeated topical dosing with eye drops, hence reducing the cost of therapy. In 2007, Li and Chauhan conducted in vitro experiments to deliver timolol maleate through the use of pHEMA-based hydrogel contact lenses [74]. The experimental results showed that at least 20% of the drug that was entrapped in the lens will enter the cornea. This bioavailability is much greater than that of conventional topical dosing through eye drops.

### 5.3. Antimicrobial Strategies

Soft contact lenses synthesized from pHEMA-based hydrogels are widely used to correct refractive errors of the eyes and for sustained drug delivery to treat ocular diseases. Despite the benefits brought about by contact lenses, they are also the causes of eye infections and inflammatory episodes due to microbial contamination. These microbially driven adverse events range from microbial keratitis (MK), infiltrative keratitis (IK), contact lens-induced peripheral ulcer (CLPU), and contact lens-induced acute red eye (CLARE) [66]. Severe ocular infections and inflammation resulting from microbial contamination of contact lenses may lead to corneal injury resulting in vision impairment.

**Figure 8:**
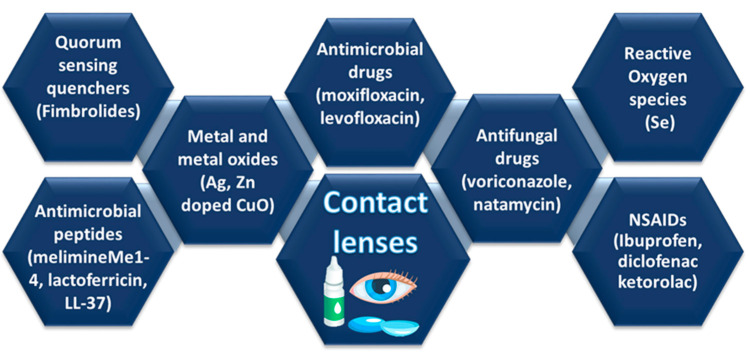
Antimicrobial strategies to fabricate microbiocidal contact lenses.

Contamination of contact lenses can be attributed to various microbes, such as bacteria, parasites, viruses, and fungi. Gram-positive bacteria, such as *streptococcus pneumoniae* and *Staphylococcus aureus*, are predominantly responsible for the prevalence of contact lens-related CLPU and IK, while Gram-negative bacteria, such as *pseudomonas aeruginosa, Haemophilus influenzae*, and *chlamydia trachomatis* are predominantly responsible for the prevalence of CLARE [67]. Since pure pHEMA does not possess antimicrobial properties against a broad range of gram-positive and gram-negative bacteria, antimicrobial approaches can be utilized to fabricate antimicrobial contact lenses to combat different microbes. These antimicrobial agents suppress or stop the growth of microbes through four modes of action: (1) penetrating directly into microbial cells, (2) modifying the microbial-substrate interfaces, (3) interfering with the quorum-sensing mechanism of microbial cells, and (4) generating reactive oxygen species [67]. Figure 8 illustrates different antimicrobial strategies for the development of antimicrobial contact lenses employing antibiotics, antifungal drugs, antimicrobial peptides, passive surface modifications, free-radical fabricating agents, quorum sensing quenchers, metals and metal oxide nanostructured materials, and coatings with antimicrobial nanomaterials.

Biomedical devices are widely used for the treatment of diseases and to replace injured or diseased organs or tissues in the human body. Since some of these devices are exposed to hostile external environments, they can potentially become contaminated with harmful pathogens and other infectious agents. This increases the possibility of developing an infection, which may lead to further complications, at the site where the biomedical device is employed. Therefore, adopting effective antimicrobial strategies is essential for the safe and effective use of biomedical devices to advance healthcare [75]. Table 2 lists the different approaches for incorporation of antimicrobial drugs into pHEMA contact lenses.

Silver is recognized for its remarkable microbicidal properties against a broad spectrum of gram-positive, gram-negative, and multidrug-resistant bacteria, protozoa, and fungi [85]. Silver-infused polymers have been employed to fabricate commercially available antimicrobial contact lens cases. Upon the addition of sterile contact lens solution to the cases, silver ions will be released into the solution at a sustained rate to kill the harmful microorganisms and to inhibit their growth [67]. The multiple biocidal actions of silver ions may also prevent contemporaneous bacterial strain resistance [66]. The biocidal silver containing-agents are due to (i) they generate reactive oxygen species; (ii) the interfering with membrane protein, enzymes, and DNA; (iii) their interaction with the bacterial cell wall [85,86,87]. Antibiotics, such as tetracycline, erythromycin, chloramphenicol, and ciprofloxacin can also be used to fabricate antimicrobial contact lenses. Antibiotics work by directly destroying or inhibiting the growth of bacteria on the contact lens and the cornea. Qin et al. loaded contact lenses with ciprofloxacin to treat *Pseudomonas aeruginosa* bacterial keratitis in an ex vivo infection model, with the study showing good efficacy and cytocompatibility with human corneal epithelial cells [88].

Other strategies to synthesize antimicrobial contact lenses include utilizing antimicrobial agents fabricated through other methods. These methods include generating reactive oxygen species, use of nonsteroidal anti-inflammatory drugs, fimbrolides (quorum-sensing quenchers), antimicrobial peptides, and passive non-cidal molecules [67,73,89,90]. While many different approaches to synthesize antimicrobial contact lenses and lens cases have been investigated, the most promising one involves using silver-infused contact lens and lens cases which are already available commercially. The other antimicrobial strategies, through continuous research, may also find commercial applicability in the near future.

## 6. Conclusions

The purpose of this review was to bring together state-of-the-art research on pHEMA and their applications. pHEMA is a biomaterial that has been utilized in many biomedical applications. pHEMA is rarely used in isolation and is usually utilized as a copolymer with other biomaterials in these applications. Initially employed in soft contact lenses due to its superior biocompatibility, unique physical and mechanical properties, the biomedical applications of pHEMA have since been extended to include hydrogel wound dressings, bone tissue regeneration, cancer therapy, ocular drug delivery, artificial skin, artificial cornea, breast augmentation, catheters, intrauterine inserts and prosthesis. However, since pure pHEMA is non-degradable, it has to be combined with other materials or modified based on the desire applications. Moreover, pHEMA does not possess inherent antimicrobial properties and so additives such as silver nanoparticles, antibiotics, and nonsteroidal anti-inflammatory drugs have to be used to give biomedical devices containing pHEMA their antimicrobial characteristics to protect against infections. The excellent biocompatibility, physicochemical, and cell-friendly properties of pHEMA also make it an attractive biomaterial in tissue engineering.

The outlook for the future of pHEMA is promising given its favorable properties and unique characteristics. pHEMA can be added to different polymers and synthesized with different approaches to produce novel biomaterials with unique properties that may be useful in different areas—cancer therapy and tissue engineering and regenerative medicine. Furthermore, promising manufacturing techniques, such as 3D printing, may allow a wider diversity of biomedical devices containing pHEMA to be fabricated and with greater precision. Three-dimensional printing also allows for more flexibility in terms of customization of biomedical devices, according to patients’ needs. With continuous research and advancement in technology, pHEMA will certainly be utilized in a wider range of biomedical applications in the near future.

## Figures and Tables

**Table 1:** Briefly summarizes the biomedical applications of pHEMA, advantages, and disadvantages.

Biomedical Applications	Advantages	Disadvantages	Reference
Bone tissue generation	Biocompatibility, excellent adhesion-promoting to other polymers, elastomeric properties	Non-degradable, requiring cross-linker	[10,13]
Wound healing	Transparency, biocompatibility	Low exudate absorbability, non-degradable, requiring cross-linker	[11,14]
Cancer therapy	Stimuli-responsive, inexpensive, easily combined with different polymers and drugs	Non-degradable, requiring cross-linker	[15,16]
pHEMA hydrogel contact lens	Inexpensive, biocompatible, abundant copolymer possibilities	protein deposition issues	[12]

**Table 2:** Various approaches and drugs used for contact lens drug delivery.

No.	Method of Drug Incorporation	Polymers/Contact Lens	Drugs Used	Inference	Ref.
1	Soaking and lyophilization	pHEMA hydrogels containing cross-linked β-cyclodextrin-hyaluronan	Diclofenac	Improved the oxygen permeability, water uptake ability, surface hydrophilicity, and flexibility	[62]
2	Soaking method	HEMA, siloxane, ethylene glycol dimethacrylate, Irgacure^®^, dimethyl acrylamide	Timolol	Reduce burst release and the timolol retention time improved, the control release kinetics and uptake of timolol improved without varying the transmittance and swelling of the contact lens.	[76]
3	Polymerization of HEMA	HEMA, mPEG-PLA micelles	Latanoprost and Timolol	Improvement in drug residence time and higher bioavailability	[77]
4	Free radical polymerization of the monomer by photoinitiation	pHEMA, chitosan nanoparticles	Dexamethasone Sodium Phosphate	Significantly enhanced bioavailability of ophthalmic drugs	[78]
5	Free radical polymerization technique	pHEMA cross linked dextrin	Ciprofloxacin hydrochloride	Excellent physical stability as carrier for ciprofloxacin up to 3 months	[23]
6	Bioinspired imprinted pHEMA-hydrogels	HEMA, zinc methacrylate, 1- or 4-vinylimidazole, and N-hydroxyethyl acrylamide	Acetazolamide or Ethoxzolamide	Remarkable improvement in the performance as controlled release system	[61]
7	Prepared by photopolymerization	pHEMA, HEMA, mono-methacrylated β-CD (mono-MA-β-CD) and trimethylolpropane trimethacrylate	Puerarin	The data demonstrate that pHEMA/β-CD hydrogel contact lenses can effectively deliver puerarin through the rabbit’s cornea	[79]
8	β-CD was grafted to the gel network and soaked in drug solution	pHEMA and GMA	Diclofenac sodium	The hydrogels with pendant β-CD are particularly useful for the development of cytocompatible drug loaded SCLs	[80]
9	By ultraviolet light polymerization film coating process	PLGA films over pHEMA	Ciprofloxacin, fluorescein	PLGA film coated over pHEMA lenses sustained the release of drug, which can be controlled by changing either the ratio of drug to PLGA or the molecular mass of the PLGA used	[71]
10	Gels prepared by living /controlled imprinting technique	HEMA, acrylic acid, acrylamide and methacrylic acid	Ketotifen, diclofenac sodium	Imprinting via living polymerization extends or delays the template release profile by two-fold over that of imprinting via conventional free-radical polymerization techniques	[81]
11	Dispersion of microemulsion in contact lenses	pHEMA	Hexadecane	p-HEMA gels loaded with a microemulsion, stabilized with silica shell are transparent and releases drugs for a period of over 8 days in vitro	[82]
12	Molecular imprinting technique	HEMA+ methacrylic acid or meth-methacrylate	Timolol	Incorporation of meth-methacrylate as comonomer increases the timolol loading capacity to therapeutically useful levels while retaining appropriate release characteristics in vitro	[83]
13	Molecular imprinting technique	HEMA + methacrylamide propyl trimethylammonium chloride	Azulene	Molecular imprinting is capable to store the anionic drug such as azulene based on ion-exchange reaction	[84]

## Data Availability

Not applicable.
